# Swedish Women at Home

**Published:** 1883-08

**Authors:** 


### SWEDISH WOMEN AT HOME.

The white or black handkerchief worn upon the heads of the women appears everywhere, and beneath the front edge of these head-dresses the hair is seen to be brushed smoothly and plainly back over noticeably broad, clear, fair brows. The women are often observed working in the fields and bearing heavy burdens; but their appearance does not present such marked and distressing evidence of premature old age, nor their forms look so crushed down and bent by toil, as in Germany. They flock to the city in search of employment, and may be often seen carrying bricks and mortar for the scanty wages of one krone per day; but such is their extreme frugality, that many of them actually accumulate out of this slender remuneration of twenty-seven cents a day a property sufficient for their old age. A gentleman told us of one energetic woman who out of such earnings saved up enough to start a small factory, which, under her frugal, wise management, has grown into a thriving business, until now this quondam hod-carrier has become a rich factory proprietor.—*Cor. Chicago Tribune.*

---

WHATEVER busies the mind without corrupting it has at least this use. that it rescues the day from idleness; and he that is never idle will not often be vicious—indeed, if wisely busy, he cannot be so.

THE ostrich farm recently started in Southern California for the production of plumes is succeeding admirably. Several of the birds are already incubating, and the place and climate chosen are eminently fit.

IF there was any truth whatever in the popular belief that dogs go mad exclusively in hot weather, there might be some justification for the dog-catching business, but it has been conclusively shown that this is not the case. Rabies in dogs is a very rare disease, most of the alleged mad dogs being victims of distemper madness—a disease painful to the dogs, but not communicable to human beings. The dogs that are peculiarly liable to rabies are the Spitz and the Newfoundland—the former having almost a monopoly of the disease in this climate. The so-called ownerless cur, unless he is a Spitz, very rarely suffers from rabies, and it is hard that he should be punished with death because of the faults of the infamous Spitz and the unhealthy Newfoundland.—*N. Y. Times.*

IT doesn't take a northern invalid very long to get well in Florida. When the first week's hotel bill is presented he generally says: "I guess I am well enough to start for home this afternoon."—*The Judge.*

